# Optical and dielectric sensors based on antimicrobial peptides for microorganism diagnosis

**DOI:** 10.3389/fmicb.2014.00443

**Published:** 2014-08-20

**Authors:** Rafael R. Silva, Karen Y. P. S. Avelino, Kalline L. Ribeiro, Octavio L. Franco, Maria D. L. Oliveira, Cesar A. S. Andrade

**Affiliations:** ^1^Programa de Pós-Graduação em Inovação Terapêutica, Universidade Federal de PernambucoRecife, Brasil; ^2^Departamento de Bioquímica, Universidade Federal de PernambucoRecife, Brasil; ^3^Centro de Análises Proteômicas e Bioquímicas, Universidade Católica de BrasíliaBrasília-DF, Brasil

**Keywords:** antimicrobial peptides, biosensors, bacterial infections, impedance spectroscopy, fluorescence spectroscopy

## Abstract

Antimicrobial peptides (AMPs) are natural compounds isolated from a wide variety of organisms that include microorganisms, insects, amphibians, plants, and humans. These biomolecules are considered as part of the innate immune system and are known as natural antibiotics, presenting a broad spectrum of activities against bacteria, fungi, and/or viruses. Technological innovations have enabled AMPs to be utilized for the development of novel biodetection devices. Advances in nanotechnology, such as the synthesis of nanocomposites, nanoparticles, and nanotubes have permitted the development of nanostructured platforms with biocompatibility and greater surface areas for the immobilization of biocomponents, arising as additional tools for obtaining more efficient biosensors. Diverse AMPs have been used as biological recognition elements for obtaining biosensors with more specificity and lower detection limits, whose analytical response can be evaluated through electrochemical impedance and fluorescence spectroscopies. AMP-based biosensors have shown potential for applications such as supplementary tools for conventional diagnosis methods of microorganisms. In this review, conventional methods for microorganism diagnosis as well new strategies using AMPs for the development of impedimetric and fluorescent biosensors are highlighted. AMP-based biosensors show promise as methods for diagnosing infections and bacterial contaminations as well as applications in quality control for clinical analyses and microbiological laboratories.

## ANTIMICROBIAL PEPTIDES

Currently, the search for novel compounds with antibiotic ability to overcome bacterial resistance has increased. Antimicrobial peptides (AMPs) appear to be an excellent alternative, since more effective therapeutic approaches are required for many types of pathogens (Brogden et al., 2005; Hancock and Sahl, 2006; Schmidtchen et al., 2014). AMPs are components of the innate immune system, acting in defense against multiple pathogens (Schmidtchen et al., 2014). In general, AMPs may also show other different activities such as antiviral or antitumor properties, making them excellent candidates as new therapeutic drugs (Reddy et al., 2004).

Antimicrobial peptides are biomolecules present in diverse organisms, such as insects, plants, and animals (Mendez et al., 1990; Schnapp and Harris, 1998). In general, AMPs are mainly cationic small molecules composed of 10–50 amino acids residues in length, with molecular masses ranging from 1 to 5 kDa. In addition, AMPs comprise a chemically and structurally heterogeneous family (Andreu and Rivas, 1998; Costa et al., 2011).

In general, AMPs only assume an amphipathic structure after interacting with the bacterial membrane, since it is not a favorable structure in an aqueous environment, allowing lethal permeabilization of the bacterial membrane (van’t Hof et al., 2001; Costa et al., 2011). The classical modes of action described previously in the literature are not necessarily exclusive of one other (van’t Hof et al., 2001; Brogden et al., 2005; Toke, 2005; Bechinger and Lohner, 2006). In the case of the barrel-stave model, a lipid bilayer disruption by AMPs occurs, until the peptides reach a threshold concentration and insert themselves across the bilayer to obtain peptide-lined pores. Subsequently, a membrane solubilization into micellar structures occurs, resulting in the carpet model or in forming peptide-and-lipid lined pores (toroidal pore model; Rapaport and Shai, 1991).

**FIGURE 1:**
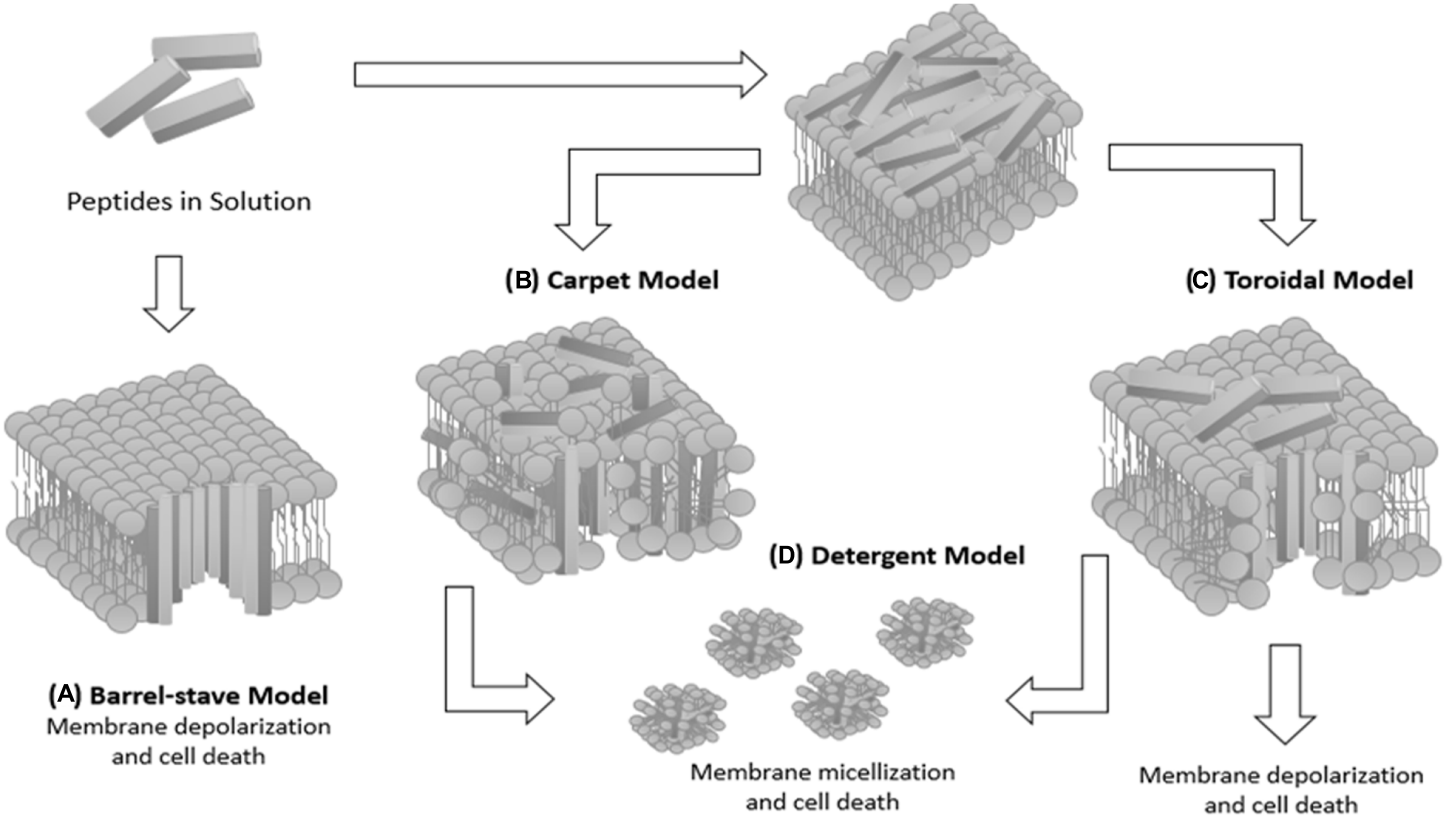
**Schematic diagram summarizing the possible mechanisms of peptide–membrane interaction. (A)** Barrel-stave model: the peptide assumes an amphipathic helical conformation and organizes itself to form a structure with a central lumen, like a barrel. The hydrophobic part of the AMP interacts with the lipid acyl chains of the membrane and their hydrophilic part is exposed so as to form the lumen of the transmembrane aqueous channel. **(B)** Carpet model: cationic peptides are attracted by anionic phospholipids covering the surface of the bacterial membrane until reaching a saturation point, resulting in solubilization of the bacterial membrane. **(C)** Toroidal model: the peptide helices are inserted parallel to the membrane and cause folding of the lipid monolayers in order to form a pore formed by peptides interspersed with phospholipids. In this model, an association between polar peptide groups with the lipid polar head groups is established. **(D)** Detergent model: membrane solubilization occurs, leading to formation of micellar structures, resulting in the destabilization of the lipid membrane and cell death.

The mechanisms of action of AMPs (**Figure 1**) were reviewed and described by Nguyen et al. (2011). In this new proposal, pore formation in the disordered toroidal pore model is more stochastic, involving low peptide quantity. The presence of peptides can directly affect the bilayer thickness and, therefore, the membrane is remodeled to form rich domains of anionic lipids on the peptide surface. In addition, AMPs can form non-bilayer intermediates in the membrane coupled to small anions. On the other hand, in the molecular electroporation model, a peptide accumulation occurs on the outer lipid membrane leaflet, making the membrane permeable to various molecules including the AMPs (Nguyen et al., 2011).

Nanotechnology has been widely introduced for multidisciplinary applications, especially associated with the use of AMPs in biology and biomedicine (Barkalina et al., 2014). Among the diverse uses of AMPs have been highlighted quality controls of foods, raising animals, controlled release systems and biosensors (Li et al., 2012; Sobczak et al., 2013). In this context, the peptide nisin has been applied as a milk-derived preservative for quality control in foods (Bi et al., 2011). In animal husbandry, peptides can be used in ruminants as an alternative to common antibiotics, avoiding bacterial resistance or assisting in the immunization of animals for the prevention of diseases (Cheema et al., 2011).

Infectious diseases have been part of humanity for centuries, some of them killing millions of people worldwide. Different types of pathogens (bacteria, viruses, fungi, and parasites) can cause infections from either exogenous (acquired from the environment, animals, or people) or endogenous (from the normal flora) origins (Mazzoni et al., 1987; Murthy et al., 2013). Furthermore, in agribusiness, the use of purified peptides, as well as the development of transgenic plants, aims to minimize plant diseases, avoiding the use of fungicides (Keymanesh et al., 2009). In clinical applications, some AMPs are in the early stages of clinical research to prevent inflammation and sepsis (Schuerholz et al., 2012).

Identification and quantification of pathogenic agents are two important parameters for the diagnosis of bacterial infections and the implementation of effective drug therapy (Lazcka et al., 2007). The most common methods are direct examination by counting bacterial colonies in culture plates and serodiagnosis tests (van Pelt et al., 1999). Direct examination by optical microscopy can identify the morphology using a simple and specific Gram staining. Culture media allow the growth as well as the isolation and identification of specific types of bacteria (Szakal and Pal, 2003; Bragulat et al., 2004). However, these methods are time-consuming and require specialized technicians. Of note, it may require weeks to obtain a correct diagnosis of some pathogens from samples of mucosa, skin, or blood (Benes et al., 1996). Unfortunately, a few days may be sufficient to significantly worsen the clinical status of the patient before receiving appropriate treatment.

Traditional methods of diagnosis also consist of molecular tests or nucleic acid amplification test (NAAT). Techniques based on NAAT include polymerase chain reaction (PCR), ligase chain reaction, transcription mediated amplification, strand displacement amplification, and loop-mediated isothermal amplification (Andersen et al., 2000; Yang et al., 2002; Palka-Santini et al., 2009). Techniques based on nucleic acids enable specific molecular detection through hybridization between a DNA molecule and its complementary strand (Junhui et al., 1997; Wang et al., 1997; Wang, 1998). Although effective, these techniques may present some limitations, such as the need for sample enrichment and purification prior to analysis, prolonged experimentation time, false-positive results due to cross-reactions, and high cost (Caygill et al., 2010; Singh et al., 2014).

In order to reduce or overcome these restrictions, new detection methods are required, and biosensors are considered promising tools for clinical diagnosis (Abdel-Hamid et al., 1999; Deisingh and Thompson, 2004). In this context, AMPs can be an excellent alternative for the development of biosensors, since their potential use for specific detection of pathogenic bacteria has been demonstrated (Kulagina et al., 2005).

## STRATEGIES FOR THE DEVELOPMENT OF AMP-BASED BIOSENSORS

Advances in nanotechnology have contributed to the improvement of biotechnological research associated with the rapid progression of biodetection devices (Jianrong et al., 2004; Fournier-Wirth and Coste, 2010; Yeom et al., 2011). Nanostructures have been extensively used in the biosensor development due to their unique physicochemical characteristics such as quantum size effect, elevated ratio between surface area and volume, and analytical signal amplification capacity (Jianrong et al., 2004; Li et al., 2010). The utilization of AMPs as recognition elements in biosensors is a relatively new concept, but their development is of great relevance due to numerous potential areas for application (Etayash et al., 2014).

**FIGURE 2:**
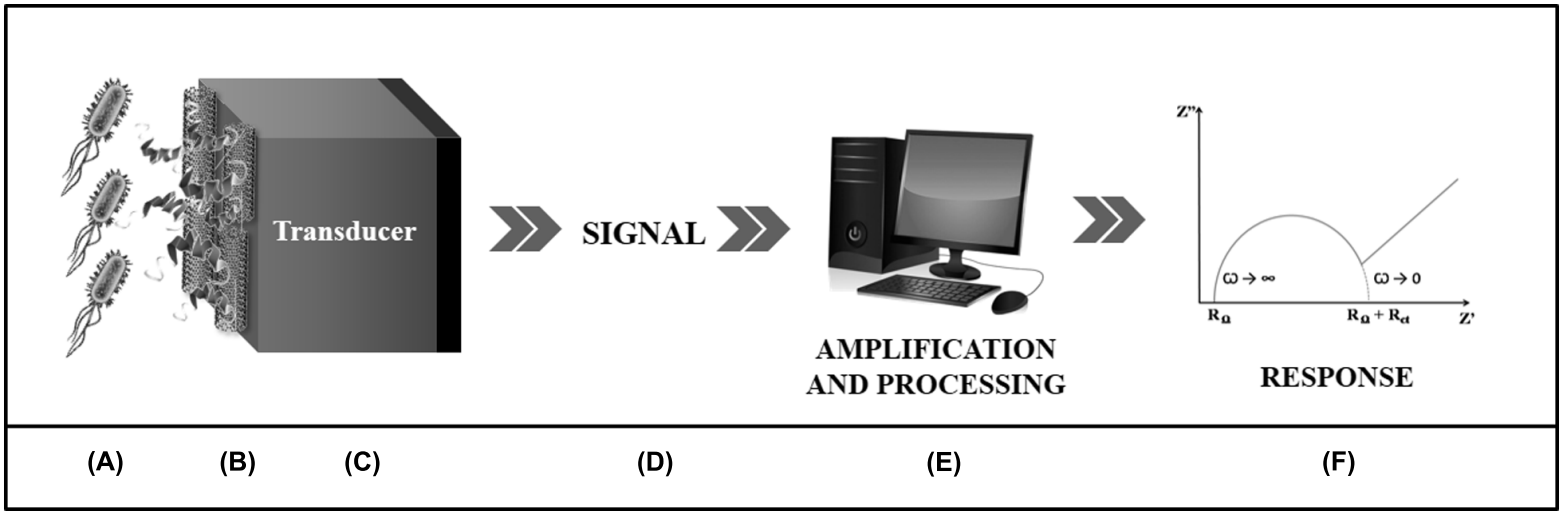
**Schematic presentation of an electrochemical biosensor.** The detection of the analyte **(A)** is performed through a biological component **(B)** associated to a transducer **(C)**. This is responsible for converting the biochemical response arising from the biospecific interaction into an electrical signal **(D)** which will be amplified and processed by a computer software **(E)** which will describe, through graphs **(F)**, the bioactivity of the system.

Biosensors are chemical devices comprising two basic functional units (**Figure 2**). The first one is a biomolecule responsible for recognition of the target substance through specific intermolecular binding or by means of catalytic reactions (D’Orazio, 2003). The second is the transducer that converts the biochemical response into a measurable electric signal, which is mainly proportional to analyte concentration (Higgins and Lowe, 1987; Hulanicki et al., 1991; Thévenot et al., 2001). In addition, diverse transducers can be used for conversion of the biochemical response into a quantifiable analytical signal, being classified according to their physical principles as electrochemical, electrical, optical, piezoelectric, calorimetric, acoustic, and magnetic (Hulanicki et al., 1991; Sethi, 1994; Marco and Barcelo, 1996; Thévenot et al., 2001).

Electrochemical impedance spectroscopy (EIS) comprises the current main strategy used for the development of biodevices based on AMPs, as shown in **Table 1**. EIS is an effective method for the investigation of modified surfaces and monitoring of interfacial processes, allowing the characterization of electrochemical systems (Stoynov and Savova-Stoynova, 1986; Bard and Faulkner, 2000; Lasia, 2002). In the EIS technique, a signal of perturbation of low amplitude, in the form of sinusoidal potential and different frequency values, is applied to the transducer, forming sinusoidal alternating current (Macdonald, 1990, 1992; Katz and Willner, 2003; Chang and Park, 2010). EIS is a valuable tool for interfacial phenomena analyses occurring between electrode and solution. Dielectric analyses allows for the evaluation of electron charge-transfer parameters, double-layer, electrical layer, and modeling the experimental data to equivalent circuits (Stoynov and Savova-Stoynova, 1986; Macdonald, 1990; Bott, 2001; Oliveira et al., 2011). EIS is a technique of great relevance due to numerous technological applications such as the development of biosensors, studies of dielectric materials, corrosion processes, biofuel cells and rechargeable batteries (Macdonald, 1992; Guan et al., 2004). EIS is a new experimental approach for understanding AMP mechanisms of action through lipid membrane properties by monitoring changes such as thickness, ion permeability and homogeneity after peptide exposure (Chang et al., 2008; Oliveira et al., 2011; Nascimento et al., 2012; Sugihara et al., 2012).

**Table 1:** Antimicrobial peptides, their immobilization substrates and molecular targets with the respective detection limits obtained through different techniques.

AMPs	Substrate immobilization	Target recognition	Detection limit	Technique	Reference
Magainin I	Gold surface – lipoic acid *N*-hydroxysuccinimide – ferrocene	*Escherichia coli* O157:H7	10^3^ CFU ⋅ mL^-1^	Electrochemical impedance	Li et al. (2014)
Magainin I	Glass microbeads – *N*-[γ-maleimidobutyryloxy] succinimide ester (GMBS) – cysteine	Non-pathogenic *Escherichia coli*	10^3^ cells ⋅ mL^-1^	Fluorescence microscopy	Yoo et al. (2014)
Magainin I	Gold surface – cysteine	*Salmonella typhimurium*	10^3^ CFU ⋅ mL^-1^	Electrical impedance	Mannoor et al. (2010)
Leucocin A	Gold surface – cysteamine	*Listeria monocytogenes*	10^3^ CFU ⋅ mL^-1^	Electrical impedance	Etayash et al. (2014)
Bactenecin	Glass slide – 3-mercaptopropyl triethoxysilane (MPTES) – GMBS	*Brucella melitensis* Vaccinia virus Venezuelan equine encephalitis virus *Coxiella burnetti*	5 × 10^4^ cells ⋅ mL^-1^ <5 × 10^5^ PFU ⋅ mL^-1^ <5 × 10^5^ PFU ⋅ mL^-1^ 5 × 10^5^ cells ⋅ mL^-1^	Fluorescence spectroscopy	Kulagina et al. (2007)
G10KHc	Gold surface – cysteine	*Pseudomonas aeruginosa*	10^5^ CFU ⋅ mL^-1^	Electrical impedance	Lillehoj et al. (2014)
C16G2cys	Gold surface – cysteine	*Streptococcus mutans*	10^5^ CFU ⋅ mL^-1^	Electrical impedance	Lillehoj et al. (2014)
Cecoprin A	Glass slide – poly(dimethyl) siloxane (PDMS)	Botulinum toxin A	1 ng ⋅ mL^-1^	Fluorescence spectroscopy	Kulagina et al. (2006)
Melittin	Glass slide – PDMS	Botulinum toxin B	10 ng ⋅ mL^-1^	Fluorescence spectroscopy	Kulagina et al. (2006)

*CFU, colony forming units; PFU, plaque forming units.

Several biological elements can be used for the manufacturing of biosensitive systems, such as cell receptors, enzymes, antibodies, antigens, nucleic acids, aptamers, lectins, and peptides (Byfield and Abuknesha, 1994; Guan et al., 2004; Caygill et al., 2010). In particular, AMPs are outstanding molecules due to their wide biotechnological applications (Dawson and Liu, 2008; Meng et al., 2010; Seo et al., 2012). In addition, AMPs are promising molecules for the development of biosensors due to the possibility of recognizing a wide variety of pathogenic agents, including bacteria, fungi, toxins, and viruses with lipoprotein envelope (Zasloff, 2002; De Smet and Contreras, 2005; Kulagina et al., 2007).

The basic principle that enables the use of AMPs in biosensors is its ability to selectively interact with the cell membrane components of pathogens (Kulagina et al., 2005). The (bio) interaction is mainly driven by electrostatic forces, hydrogen bonds, and hydrophobic interactions (Seelig, 2004). Biological recognition thermodynamics depends on lipid membrane composition and peptide properties such as hydrophobicity, amphipathicity, molecular charge, and degree of secondary structure angle (Mozsolits et al., 2001; Papo and Shai, 2003; Fernandes et al., 2012; Oliveira et al., 2013). Therefore, AMP specificity is based on the different molecular affinities, allowing their employment in diagnostic tests (Kulagina et al., 2005; Etayash et al., 2014; Li et al., 2014).

Interdigitated electrodes are an excellent alternative for biosensor development. Mannoor et al. (2010) reported the development of an interdigitated capacitive biosensor for detection of *Escherichia coli* and *Salmonella typhimurium*. In this study, magainin I was immobilized on gold microelectrodes via its *C*-terminal cysteine residue, and the binding capacity to bacterial cells was evaluated by EIS. The changes in the dielectric properties of the modified electrode surface allowed the determination of selectivity for pathogenic Gram-negative bacteria *E. coli* and *Salmonella typhimurium* in relation to non-pathogenic *E. coli* and the Gram-positive species *Listeria monocytogenes* (Mannoor et al., 2010).

Recently, Li et al. (2014) developed an impedimetric biosensor with magainin I conjugated to a structured film of ferrocene for bacterial detection. The biosensor showed a preferential selectivity for *E. coli* O157: H7 followed by non-pathogenic *E. coli* K12, *Bacillus subtilis* and *Staphylococcus epidermidis*. In addition, a detection limit was observed for *E. coli* O157: H7 of 10^3^ CFU mL^-1^.

A microelectromechanical sensor using two synthetic peptides (C16G2cys or G10KHc) was developed for the detection of *Streptococcus mutans* and *Pseudomonas aeruginosa* (Lillehoj et al., 2014).It is important to note that C16G2cys and G10KHc have a cysteine amino acid residue in the *C*-terminus, and the -SH group of the cysteine can be used to promote binding on the gold surface, resulting in the vertical orientation of the recognition molecules.

Among the optical properties explored for the construction of biosensors, one of the most prevalent is fluorescence, evaluated by a significant number of techniques that allow the analysis of the systems conjugated with fluorophores and molecular targets quantification (Gauglitz, 2005; Altschuh et al., 2006; Lazcka et al., 2007; Fan et al., 2008). Fluorescence is a phenomenon of photon emissions, resulting from the passage of a valence electron in an orbital of ground state to an orbital of higher energy to the absorption of radiation of appropriate wavelength. Upon returning to the state of origin, photons are liberated with bottom energy to absorbed light. Fluorescence spectroscopy allows for the quantification of the analyte with sensibility, low cost, and easy implementation (Eaton, 1990; Lazcka et al., 2007). Thus, the molecular recognition is assessed through variations in fluorescence properties after the interaction of the bioreceptor with the specific target (McFarland and Finney, 2001; Altschuh et al., 2006).

In this context, magainin I was immobilized on glass slides modified with 3-mercaptopropyl triethoxysilane (MPTES) and *N*-(γ-maleimidobutyryloxy) succinimide ester (GMBS) through direct covalent binding or by avidin–biotin coupling, being used as a recognition molecule for *E. coli* O157: H7 and *Salmonella typhimurium* (Kulagina et al., 2005). The immobilization method interferes with biosensor sensitivity, and the direct binding of magainin I reduces non-specific interactions, resulting in detection limits of 1.6 × 10^5^ and 6.5 × 10^4^ cells mL^-1^ for *E. coli* and *Salmonella typhimurium* labeled with Cy5, respectively. Through the indirect method values of 6.8 × 10^5^ and 5.6 × 10^5^ cells mL^-1^ were obtained (Kulagina et al., 2005). Moreover, cecropin A, parasin, magainin I, and polymyxin B and E, immobilized on a glass slide modified with MPTES and GMBS, were also used in screening fluorescent assays for detection of *E. coli* O157:H7 and *Salmonella typhimurium* (Kulagina et al., 2006). The assay in “sandwich” format, with the employment of Cy3-labeled anti-*E. coli* or anti-*Salmonella,* demonstrated different AMP affinities for pathogenic species (Kulagina et al., 2006). In another study, cecropin (A, B, and P), parasin, magainin I, polymyxin (B and E), melittin, and bactenecin were evaluated for the biodetection of viral particles and bacterial cells using Cy3 (Kulagina et al., 2007).

Cecropin P1 (CP1), SMAP-29, and PGQ were used as alternative molecules for detection of *E. coli* O157:H7 in substitution of the *anti-E. coli* O157:H7 antibody (Arcidiacono et al., 2008). Through screening in solution and quantification of fluorescence, it was verified that the detection limits for Cy5 CP1, Cy5 SMAP, and Cy5 PGQ were 10^4^, 10^5^ e10^6^ CFU mL^-1^, respectively, in comparison to 10^5^ CFU mL^-1^ for Cy5 anti-*E. coli* O157 antibody. Because of the high sensitivity of the CP1 and specificity of the anti-*E. coli* O157:H7 antibody, a prototype immuno-magnetic bead biosensor was developed, resulting in a 10-fold improvement in sensitivity (Arcidiacono et al., 2008).

Glass microspheres (GMs) based on microfluidic chip were used for magainin I immobilization as a new method for *E. coli* biodetection (Yoo et al., 2014). GMs provided greater detection efficiency through the increase of superficial area of adsorption relative to the volume, enabling a greater number of bonds between microorganism-AMPs. The biodevice presented a good detection efficiency of 87% in a limit of 10^3^ cells mL^-1^ obtained by image analysis under fluorescence microscopy (Yoo et al., 2014).

The most common application of AMP-based biosensors is the identification of diverse bacteria such as *E. coli*, *Salmonella typhimurium*, *L. monocytogenes*, *Streptococcus mutans*, *P. aeruginosa*, and others (Mannoor et al., 2010; Li et al., 2014). In this scenario, AMPs stand out for presenting differential characteristics and large applicability potential, since AMPs are highly stable under unsuitable conditions and can be continuously exposed to natural surroundings (Casteels et al., 1989; Mannoor et al., 2010; Laverty et al., 2011; Hassan et al., 2012; Dawgul et al., 2014). AMPs are capable of interacting with invariant components of the target surfaces (Kulagina et al., 2006), providing each peptide with the possibility of recognizing a variety of pathogens (Zasloff, 2002; De Smet and Contreras, 2005; Kulagina et al., 2007). Despite the importance of bacterial diagnosis, diverse studies have undertaken to develop detection systems for other microorganisms and toxins (Zasloff, 2002; De Smet and Contreras, 2005; Kulagina et al., 2007).

Therefore, AMPs can be used in nanostructured platforms for the detection of pathogenic agents due to their singular properties, ease of acquisition, and relevant biological activity (Kulagina et al., 2005; Yonekita et al., 2013). AMPs are an excellent alternative for obtaining new, sensitive, inexpensive, portable, versatile, and fast methods of diagnostics for analyte identification and quantification (Caygill et al., 2010; Yoo et al., 2014). Thus, nanobiosensors based on AMPs can be used in basic and applied research, clinical analysis, commercial applications, microbiological quality control, and environmental monitoring (Higgins and Lowe, 1987; D’Orazio, 2003; Mehrvar and Abdi, 2004).

## CONCLUSION

Antimicrobial peptides are considered promising molecules in the development of biodetection devices. Nanotechnology has enabled the construction of biosensors based on AMPs with more specificity and sensitivity for detection of pathogenic agents. Biosensors based on AMPs are a useful tool since it is possible to investigate a wide variety of molecular targets in real time, providing quantitative or semi-quantitative analytical information that is both specific and selective. In spite of AMP-based biosensors being prototypes and their remaining challenges to commercialization such as reproducibility, validation and proper standardization, they may be considered valuable alternatives for obtaining new diagnostic methods.

## Conflict of Interest Statement

The authors declare that the research was conducted in the absence of any commercial or financial relationships that could be construed as a potential conflict of interest.
